# 47, XXX syndrome with infertility, premature ovarian insufficiency, and streak ovaries

**DOI:** 10.1002/ccr3.2207

**Published:** 2019-05-14

**Authors:** Munazzah Rafique, Solaiman AlObaid, Dania Al‐Jaroudi

**Affiliations:** ^1^ Women Specialized Hospital King Fahad Medical City Riyadh Saudi Arabia; ^2^ Reproductive Endocrinology & Infertility Medicine Department King Fahad Medical City Riyadh Saudi Arabia

**Keywords:** premature ovarian failure, primary infertility, streak ovaries, Trisomy X

## Abstract

We report a patient with primary infertility and clinical manifestation of premature ovarian insufficiency (POI) who upon investigation was found to have streak ovaries, and genetic testing revealed Trisomy X (47, XXX). Therefore, we suggest for genetic testing in women with POI to detect common aneuploidies for better counseling and treatment.

## 1 INTRODUCTION

There are many questions in trisomy X that are yet not answered, as this genetic disarray has received very little attention by clinicians. Jacobs et al had reported triple X syndrome (47, XXX) as the “super female” in 1959.^1^, ^2^ It is a sex chromosome aneuploidy in which females have an additional X chromosome, in contrast to 46, XX karyotype in typical females.^1^ It is a rare chromosomal abnormality, occurring in approximately one in 1000 female births.^2^ Initially, clinodactyly and epicanthal folds were described as minor anomalies in some of the cases, along with congenital heart diseases.^3^ In the 1980s, further reports on women with an extra X chromosome showed specific facial dysmorphism, various minor hand and feet anomalies, clubfoot, heart anomalies, strabismus, and genitourinary malformations.^4^

Trisomy X syndrome is mainly derived from maternal nondisjunction errors during meiosis.^5^ Currently, it is considered that only 10% of individuals with trisomy X are diagnosed.^5^ Advanced maternal age is a significant risk factor of triple X.^5^ In theory, it was hypothesized that there might be an increased risk of sex chromosome aneuploidy in the offspring of these women.However, the pedigree study showed normal karyotype of the children who had no menifestations of 47, XXX syndrome.^6^, ^7^

There is a significant disparity in the phenotype, with relatively subtle and nonspecific physical characteristics. Some individuals present with mild symptoms, whereas others exhibit more significant physical and psychological features.^1^, ^2^ Most of the girls born with triple X chromosomes have no signs or symptoms at birth.^8^ Their height tends to be variable as reported short stature in some report but mostly higher than average. There is a slight lowering of mean IQ, but no specific abnormal behavioral patterns are observed.^1^, ^2^, ^6^, ^9^ Some individuals may exhibit seizures, developmental delays (speech and motor), learning or intellectual disability, attention‐deficit/hyperactivity disorder (ADHD), anxiety, mood disorders, or other psychiatric symptoms.^10^, ^11^ The physical features include epicanthal folds, hypotonia, and clinodactyly along with renal and genitourinary abnormalities.^2^, ^5^, ^8^ However, fertility is usually normal.^1^, ^2^, ^3^, ^4^, ^5^, ^6^

The diagnosis of 47, XXX syndrome is made by chromosome analysis, either through cytogenetic karyotype testing or through microarray. The majority of girls with triple X syndrome do not seek medical attention and do not have had their chromosomes tested.^6^ The condition often remains undiagnosed until adulthood when the genetic defect is discovered for other reasons like infertility. Patients presenting with developmental delay, hypotonia, learning disabilities, emotional or behavioral difficulties, or dysmorphic face are diagnosed earlier usually in childhood.^9^, ^11^ Occasionally, it is found in DNA sample collected for the evaluation of other genetic polymorphism studies without signs of the physical syndrome in healthy children.^7^, ^9^ Laboratory studies including PCR amplification, X‐STR genotyping, G‐banding karyotyping study, and the pedigree study are employed to find out the possibility of mosaicism (45, X0/47, XXX) and ascertain 47, XXX karyotype without mosaic.^7^ Differential diagnosis before definitive karyotype results includes Turner syndrome (45, X0) or mosaic Turner (45, X/46, XX), trisomy X (47, XXX or mosaic), fragile X premutation, tetrasomy X, and pentasomy X.^5^ Genetic counseling is recommended so that early intervention therapies can be implemented as needed.^5^

The objective of our report is to describe an unusual phenotype of the trisomy X syndrome who was referred to as a case of primary infertility for in vitro fertilization (IVF) treatment. She underwent investigations and genetic analysis and was managed with hormonal replacement therapy and possible IVF. Her investigations showed high FSH levels, and on ultrasound, there were streak ovaries with a normal uterus. The initial diagnosis was premature ovarian insufficiency (POI) for the unknown reason. However, the genetic analysis revealed 47, XXX syndrome.

## 2 CASE PRESENTATION

**Figure 1:**
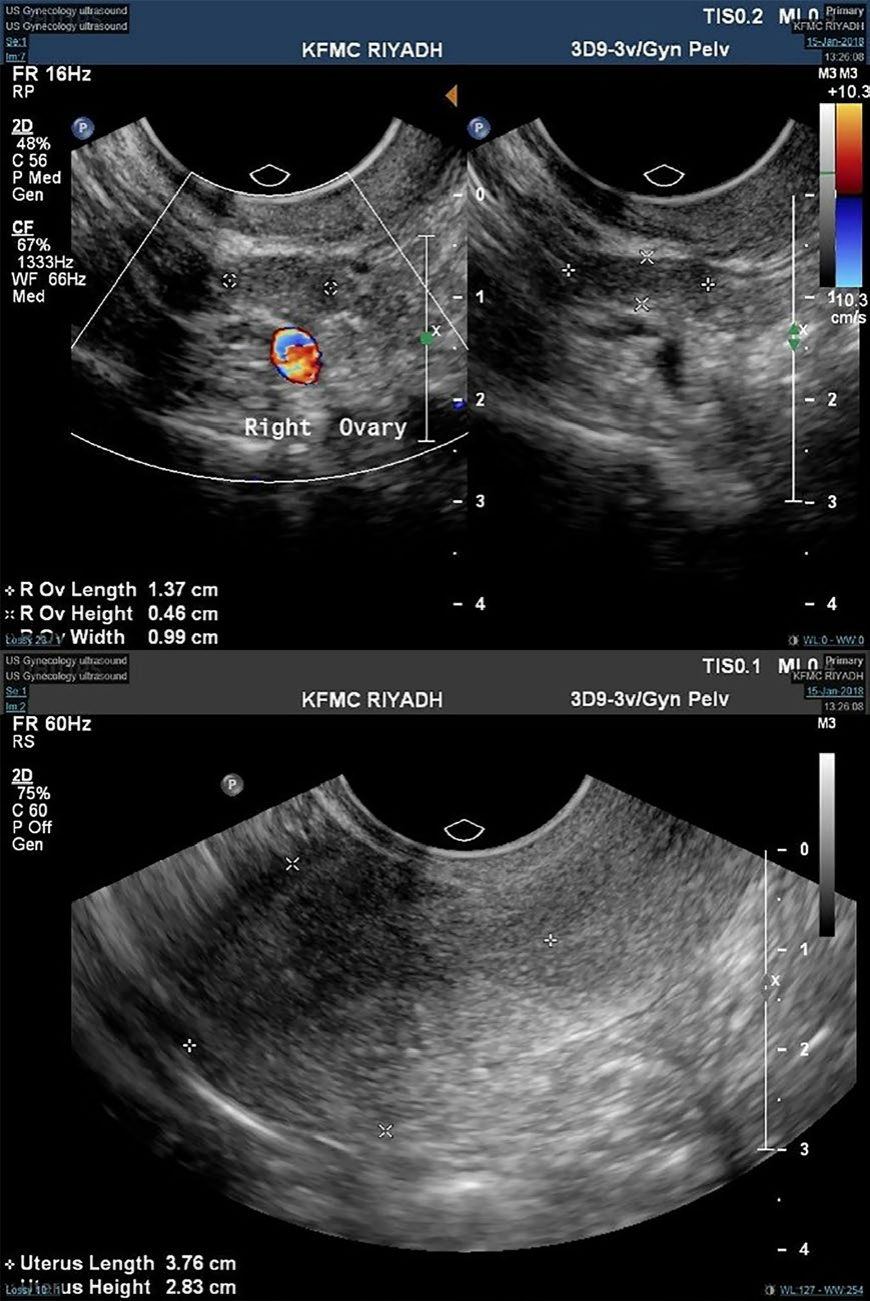
Ultrasound showing streak ovaries with no follicles and a normal uterus

The patient was referred to the reproductive endocrine and infertility medicine department as a 31‐year‐old nulliparous woman, married for five years with primary infertility for in vitro fertilization (IVF). She had a history of irregular periods mainly oligomenorrhea with prolonged irregular menstruation. She had no acne, hirsutism, weight gain, or symptoms of PCOS. She reported no hot flashes or night sweating. She was not having diabetes, thyroid problem, or other immunological problems. As childhood, she did not have the intellectual delay or behavioral problems. She had no family history with a similar condition. Her mother's age at the time of her birth was unknown. Her general examination was normal. Her BMI was 23.7 kg/m^2^. She was tall, with a height of 170 cm and an arm span of 173 cm. Hands showed nevi on the outer aspect of the left side. Teeth and feet were normal. The thyroid examination was normal. However, there was a loud mid‐diastolic murmur upon examination of the cardiovascular system. She had reported easy bruisability and eosinophilia. Her investigations revealed a hemoglobin 12.50 g/dl (11‐16g/dL), platelet count 276.0 10 × 9/L (155‐435/L), serum prolactin 10.4 ng/mL (5.18‐26.53), serum follicular hormone (FSH) 18 IU/L, repeated FSH 26.72 IU/L (3.03‐8.08 IU/L), serum luteal hormone (LH) 14.8 IU/L, serum estradiol (E2) 229 pmol/L, serum vitamin D 25‐OH (total) 38.7 nmol/L (75 ‐ 350), serum TSH 2.035 mIU/L (0.35‐4.94), serum T4 free 12.1 pmol/L (9‐19), and serum T3 free 3.8 pmol/L (2.6‐5.7). Pelvic ultrasound of the uterus was normal for size, shape, and texture, and endometrium of 3 mm and both ovaries were seen; right ovarian volume was 0.33 mL, and the left ovarian volume was 1.15 mL; and the antral follicle count was zero in both ovaries. **(**Figure 1 **)** The patient underwent diagnostic laparoscopy for tubal patency that showed normal uterus with bilateral streak ovaries and patent tubes.

Karyotype analysis revealed a female karyotype with an additional X chromosome. Cytogenetic test was consistent with triple X syndrome, and FISH analysis revealed a female genotype, an X (FISH) was carried out using the CEPX (DXZ1) and Y(SRY) DNA probe panel (Abbott, USA) to screen for the copy number of chromosomes X and Y and the presence of the SRY gene region. A total of 500 interphase nuclei were scored for each chromosome, and the signal pattern revealed three copies for the X chromosome (DXZ1). FISH signal pattern confirmed the presence of three copies of the X chromosome, which was also consistent with triple X syndrome.

The patient was started on a noncontraceptive HRT regime and calcium supplement. Her echocardiography was done and reviewed by a cardiologist who cleared her from any cardiac anomalies. Genetic counseling and evaluation by a clinical geneticist were done. Consequently, she had ovulation induction with high doses of both recombinant human follicle stimulating hormone 300 IU (rFSH (follitropin alpha, Merck Serono, Gonal‐F)) and 150 IU of human menopausal gonadotropin (HMG, Merional, IBSA). Subsequently, there was no response to treatment and eventually no pregnancy.

## 3 DISCUSSION

Triple X syndrome is uncommon in origin, and the extra X chromosome is mostly derived from maternal nondisjunction in oogenesis.^2^ There is one X chromosome that is activated, and the other two are inactivated to Barr bodies.^5^ Advanced maternal age and deviant recombination are risk factors of the syndrome.^12^ The variable phenotypic abnormalities are related to the overexpression of the genes situated on the extra X chromosomes that escape X‐inactivation.^5^ The representation of the different symptoms also contributes to the high rate of misdiagnosis. Triple X females tend to be moderately tall with final height ranging from 1 to 3 SDS of the average population.^13^ The excessive copies of the gene prolong the period of growth. Also, the alteration of the noninactivated region and hormone factors might contribute to the height increase and^12^ this was similar to our case. Some studies reported that 50% of 47, XXX females had delayed motor development and poor language skills^6^, ^10^ that was not the case in our patient since she had normal development and normal linguistic skills.

Previous literature showed that 47, XXX females usually had high levels of estrogen and progesterone, causing menstrual disorder and sexual precocity.^14^ In our case, the woman had menstrual irregularities, mainly oligomenorrhea. Moreover, her hormonal tests showed that the level of FSH and LH were all above the normal range. The abnormal level of gonadal hormone in our case was probably due to the existence of the extra X chromosome and the expression of genes that had escaped X‐inactivation but did not reach menopausal range.

Identification of a patient with 47, XXX on ultrasound is difficult, and prenatal diagnosis via amniocentesis, chorionic villi sampling, or postnatal karyotype analysis is not routinely performed.^4^ In this case, the patient and other family members were phenotypically normal, and the suspicion of syndrome was delayed until presentation for infertility. Therefore, the karyotype of 47, XXX was an unexpected finding. It is true that the condition often remains undiagnosed through adulthood, though, if the diagnosis is made in adulthood, it is most commonly due to fertility problems. The unique aspect of this case was the identification of streak ovaries as well. However, genetic tests were not performed on the ovarian tissue due to the lack of evidence to support the diagnosis and its impact on the residual ovarian tissue that may affect the spontaneous pregnancy. Performance of gNIPT for detection of sex chromosome aneuploidies including 47, XXX syndrome has a sensitivity of 91.9%‐93.8% and the specificity of 99.5%‐99.6%.^15^ Therefore, the molecular identification is usually not performed as it is not cost effective and do not change the prognosis or course of the disease process. In our patient also, standardized FISH was performed and molecular identification was not done.

The other genetic cause of POI is the fragile X mental retardation 1 (*FMR1*) gene that is located on the q arm of the X chromosome. Fragile X syndrome is due to inherited triplet repeat mutation in the *FMR1* gene and leads to familial mental retardation. POI develops in females with a premutation in the *FMR1* gene and ranges between 14% and 20% of women with familial POI and ranges between 2% and 5% of women with isolated POI.^16^, ^17^

Despite having minimal risk of transmission to the offspring and due to the spontaneous expansion of the trinucleotide repeat region, patients with POI should be tested for the FMR1 premutation. Patients with POI and known family history of genetic disorders should be tested for other rare genetic defects.^16^

The genetic causes of POI range between 20% and 25%. The size of the primordial follicle pool might be affected by genetic influence, which later leads to follicular atresia, and thus menopause.^16^ POI can be the result of X‐chromosome aberrations, and this may be due to mutations on the loci of the long arm (q) of the X chromosome. X chromosome regulates germ cell development. POI might also be a result of complete or partial monosomy X (45, XO) as in Turner syndrome.^18^ The most frequent chromosome constitution is 45, XO or complete absence of one X chromosome; the ovaries have few or no oocytes and are streak with fibrous stromal tissue.^16^, ^18^

Therefore, in the due event of having a patient presenting with POI, we recommend genetic testing to detect the three common etiologies: Turner syndrome (45, X0) or mosaic Turner (45, X/46, XX), trisomy X (47, XXX or mosaic), and fragile X premutation.

## 4 CONCLUSION

We conclude that 47, XXX females can present with primary infertility and clinical manifestation of premature ovarian insufficiency (POI). Infertility treatment is a challenge that necessitates multidisciplinary management by a reproductive endocrinologist, geneticist, psychologist, and psychiatrists when treating these patients. Moreover, the choice of infertility treatment for the patient needs to be elucidated in future studies.

## CONFLICTS OF INTEREST

None to declare.

## **AUTHOR** **CONTRIBUTIONS**

RM: compiled the endocrine data and authored the manuscript. SA: coauthored and revised the manuscript and did proofreading. JD and SA: coauthored the manuscript and performed the literature search. JD: served as the physician principally responsible for the care of the patient, and reviewed and proofread the final manuscript. All authors were directly or indirectly involved in the care of the patient.

## ETHICAL APPROVAL

IRB approval: IRB approval was obtained before proceeding to study. Consent: Informed consent was obtained from the patient for publication of this case report.
